# How to: Basics der Schrittmacherprogrammierung

**DOI:** 10.1007/s00399-022-00864-w

**Published:** 2022-05-23

**Authors:** V. Johnson, C. Israel, J. Schmitt

**Affiliations:** 1 Med. Klinik I, Abteilung für Kardiologie, UKGM Gießen, Universitätsklinik Gießen, Klinikstr. 33, 35392 Gießen, Deutschland; 2 Klinik für Innere Medizin, Kardiologie, Diabetologie und Nephrologie, Ev. Krankenhaus Bielefeld, Bielefeld, Deutschland

**Keywords:** Aktive Rhythmusimplantate, Programmierung, His-Bundle-Stimulation, Frequenzadaptation, Herzschrittmacherkontrolle, Cardiac implantable electronic devices, Programming, His-bundle pacing, Rate response, Pacemaker follow-up

## Abstract

Die Programmierung von Schrittmachersystemen, insbesondere von Zweikammerschrittmachersystemen kann die untersuchenden Ärzte vor große Herausforderungen stellen. Eine genaue Kenntnis der zu programmierenden Parameter sowie der herstellerspezifischen Algorithmen ist essentiell. Bei der Programmierung sollte darauf geachtet werden, die Programmierung den individuellen Bedürfnissen der Patient:innen anzupassen und „Out-of-the-box“-Programmierungen zu vermeiden. Ein weiteres wichtiges Ziel der Programmierung ist es, unnötige Stimulation im rechten Ventrikel zu vermeiden und dem Patienten eine gute Belastbarkeit zu ermöglichen sowie zu vermeiden, dass er eine Stimulation wahrnimmt. Algorithmen der Hersteller können hierbei helfen, müssen jedoch verstanden und bei inadäquatem Verhalten ggf. deaktiviert werden.

## Abstract (en)

Programming of implantable cardiac devices, especially dual-chamber pacemakers, can be challenging in daily clinical practice. Precise knowledge of programmable parameters is important; furthermore, one should also be familiar with the specific algorithms of each manufacturer. During programming, the patient’s individual requirements should be taken into account, but out-of-the-box programming should be avoided. Another important goal of programming should be to stimulate as much as needed but as little as possible to provide the patient good exercise capacity while not being aware of the pacing. Manufacturers’ algorithms can help reach these aims but need to be understood and—in case of inappropriate behavior—to be deactivated.

## Hinführung

Jährlich werden in Deutschland etwa 100.000 Operationen an Herzschrittmachern (HSM) durchgeführt [4]. Viele Patient:innen in Deutschland und weltweit sind mit einem Herzschrittmacher versorgt. Es ist daher von großer Bedeutung, die Grundlagen der Programmierung zu verstehen, um Fehlfunktionen zu vermeiden.

Das Ziel einer „guten“ Programmierung eines Herzschrittmachersystems soll sein, dass der Patient den Herzschrittmacher im Alltag möglichst wenig „spürt“, gut belastbar ist, und durch die gewählte Programmierung die Batterielaufzeit möglichst lang ist, um frühzeitige Aggregatwechsel zu vermeiden.

## Grundlagen der Herzschrittmachertherapie

In dem folgenden Artikel sollen die Grundzüge der Programmierung von Ein- und Zweikammerschrittmachersystemen vorgestellt werden. Ein Exkurs am Ende des Artikels beschreibt die Besonderheiten bei der Programmierung einer His-Bundle-Elektrode. Eine Übersicht über die Indikationen zur Herzschrittmachertherapie sowie die technischen Aspekte der Implantation wird an dieser Stelle auf die entsprechenden Publikationen der Europäischen Gesellschaft für Elektrophysiologie verwiesen [3, 6]. Eine Übersicht zur strukturierten Nachsorge von aktiven Rhythmusimplantaten kann hier gefunden werden [13].

Dieser Artikel hat keinen Anspruch auf Vollständigkeit und ersetzt weder ein Lehrbuch oder einen Herzschrittmachersachkundekurs.

Um die grundlegenden Einstellungen der Schrittmacherprogrammierung erklären zu können, müssen vorher die einzelnen Begriffe und einzustellenden Parameter verstanden werden. Wichtig ist auch die Kenntnis des NBG-Buchstabencode ([1], Tab. 1 sowie die Abkürzungen der Refraktär- und Blanking-Zeiten, Tab. 2).

**Table 1:** 

Ort der Stimulation	Ort der Wahrnehmung	Stimulationsart	Sensorfunktion
A: atrial	A: atrial	I: inhibiert	R: aktiviert
V: ventrikulär	V: ventrikulär	T: getriggert	–
D: dual (A + V)	D: dual (A + V)	D: dual	–
0: aus	0: aus	0: aus	–

**Table 2:** 

	Vorhof	Kammer
Stimulationsmodi	AAI, DDD	DDD, VVI, VAT
Refraktärzeiten	PVARP: postventrikuläre atriale Refraktärzeit TARP: totale atriale Refraktärzeit	VRP: ventrikuläre Refraktärzeit
Ausblendzeit („blanking“)	PVAB: postventrikuläres atriales „blanking“	PAVB: postatriales ventrikuläres „blanking“

*A* Atrium, *D* Dual (A+V), *I* Inhibiert, *V* Ventrikel, *R* Sensorfunktion aktiviert

Das Ziel jeder Programmierung eines aktiven Rhythmusimplantats sollte sein, so sicher und effektiv wie möglich zu arbeiten unter Berücksichtigung der patientenindividuellen Indikation und der maximalen Batterielaufzeit. Patient:innen mit implantierten Herzschrittmachersystem sollten nach erfolgter Herzschrittmacherimplantation und unauffälliger Kontrolle nach Entlassung einmalig nach 2–12 Wochen nach Implantation sowie danach alle 6–12 Monate nachgesorgt werden [6].

Seit Jahren ist bekannt, dass eine hohe Stimulation einer im rechtsventrikulären Apex liegenden Schrittmacherelektrode über die Zeit zu einer Verschlechterung der linksventrikulären Funktion führen kann, eine sog. „Pacemaker-induzierte“ Kardiomyopathie und RV-Stimulation deswegen weitestgehend vermieden werden sollte [10, 14]. Aus diesem Grund haben die Hersteller von aktiven Rhythmusimplantaten verschiedene Algorithmen entwickelt, um bei entsprechend vorhandenem Kammerrhythmus die ventrikuläre Stimulation weitestgehend reduzieren zu können. Diese Algorithmen finden überwiegend bei Zweikammerschrittmachersystemen ihre Anwendung.

Einkammerschrittmacher sind bei permanentem Vorhofflimmern indiziert, hier gibt es häufig wenig zu programmierende Parameter [6].

Bei Zweikammerschrittmachern gibt es nicht nur numerisch mehr zu programmierende Parameter, durch das Vorhandensein von zwei Elektroden (im rechten Atrium und rechten Ventrikel) gibt es auch die Möglichkeit der Interaktion – des „cross-talks“. Dies kann u. U. auch zu Fehlfunktionen und Problemen führen und sollte durch die sorgfältige Programmierung weitestgehend ausgeschlossen werden.

## Programmierung von Herzschrittmachern

Nach durchgeführter Bestimmung der Messwerte (Impedanz, „Sensing“ sowie der Reizschwelle) sollte eine Anpassung der programmierbaren Parameter (Output und Wahrnehmung) erfolgen. Dies erfolgt automatisiert mit tagesaktueller Anpassung der Geräte, wenn entsprechende Automatismen implementiert sind.

Da nicht alle Geräte automatische Reizschwellenmessungen durchführen können, muss bei diesen eine manuelle Messung und anschließende Adjustierung erfolgen. Zur manuellen Einstellung des Output wird häufig die doppelte Spannung der ermittelten Spannung an der Reizschwelle als Output programmiert (Beispiel: gemessene Reizschwelle 0,5 V@0.4 ms → Programmierung 1,0 V@0,4 ms; [13]). Vor dem Hintergrund einer möglichst langen Laufzeit sollte immer bedacht werden, dass eine zu große Sicherheitsmarge sich negativ auf die verbleibende Batteriespannung auswirkt und somit zu einer deutlichen Verkürzung der Batterielaufzeit mit frühzeitigem Aggregatwechsel führen kann. Daher sollte die programmierte Amplitude aus subjektiven Sicherheitsbedenken nicht zu hoch programmiert werden und die gemessene Batteriespannung möglichst nicht übersteigen.

Lediglich in den ersten 3 Monaten nach Implantation wird ein etwas höherer Wert empfohlen, falls es im Rahmen der Einheilungsphase zu einem Anstieg der Reizschwelle kommt.

Nach Programmieren des Outputs sollte auch immer ein Blick auf die voreingestellte bzw. programmierte Empfindlichkeit erfolgen. Hierbei ist es wichtig, dass alle Signale (Vorhof- und Kammer) sowohl im Sinusrhythmus als auch beim Auftreten von Rhythmusstörungen erkannt werden. Eine zu unempfindliche Einstellung der Sensitivität kann störende Signale ausblenden, aber auch dazu führen, dass Rhythmusstörungen (z. B. Vorhofflimmern) nicht richtig erkannt werden. Als Faustregel gilt in etwa, eine Empfindlichkeit von 0,5 mV im Vorhof und 2,0 mV im Ventrikel. Dies ist aber auch von den aktuell gemessenen Sensing-Werten abhängig und muss ggf. Im Verlauf angepasst werden.

Weitere zu programmierende Parameter sind der Modus und die Grundfrequenz. Dies richtet sich nach der zugrunde liegenden Indikation zur Therapie mit einem Herzschrittmacher sowie nach den induviduellen Gegebenheiten bzw. Vorlieben des Patienten. Dies betrifft auch die Aktivierung eines Aktivitätssensors, um einen situationsbezogenen Frequenzanstieg bei Bedarf zu ermöglichen.

Die Programmierung eines Einkammerschrittmachers erfolgt im VVI (R)-Modus mit einer Grundfrequenz von 50/min–60/min.

**Figure 1:**
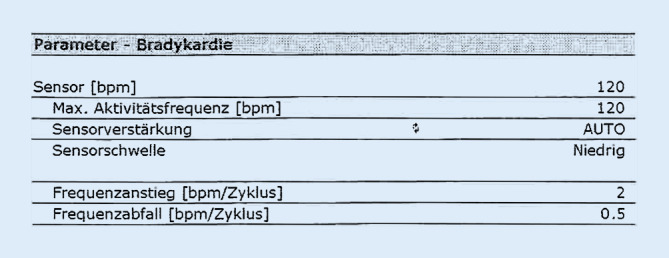

Im Rahmen der Nachsorge sollte das Herzfrequenzprofil kritisch durchgesehen werden, um zu prüfen, ob eine entsprechende Anpassung des Aktivitätssensors vorgenommen werden sollte. Eine Anamnese mit der Frage nach körperlicher Aktivität oder Belastbarkeit ist hier ebenfalls sehr hilfreich. Die Möglichkeiten der Einstellung des Aktivitätssensors sind bei allen Herstellern unterschiedlich mit verschiedenen Möglichkeiten bzw. Adjustierungen. Auch die technischen Grundlagen des Sensors (Bewegung, Akzeleration oder Atemminutenvolumen) sind unterschiedlich, unterliegen aber mechanischen Bedingungen. Weitere Unterschiede bestehen in der Empfindlichkeit des Anspringens sowie im Ausmaß in der Steigerung der Herzfrequenz. Ein zu aggressiv eingestellter Aktivitätssensor (R-Sensor) kann zu unangenehmem Herzrasen und Unwohlsein der Patient:innen führen und muss daher mit Fingerspitzengefühl eingestellt werden. Nicht selten wird nicht beim ersten ambulanten Besuch die perfekte, individuelle Einstellung gefunden. In Abb. 1 sind beispielhaft die einzustellenden Parameter abgebildet. Einen technisch anderen Ansatz verfolgt die Closed-loop-Stimulation (CLS), um auch neurohumorale Einflüsse in die Anpassung der Herzfrequenz einfließen zu lassen [11].

## Grundlagen der Programmierung eines Zweikammerschrittmachers

Zweikammerschrittmachersysteme haben deutlich mehr Möglichkeiten der Programmierung als Einkammersysteme. Das Ziel der Programmierung sollte sein, die physiologischen Leitungszeiten weitestgehend zu imitieren und ventrikuläre Stimulation, wenn möglich, zu vermeiden.

Ein wichtiger Aspekt bei der Programmierung ist das Vorhandensein einer permanenten oder intermittierenden Bradykardie und die hier angepasste Programmierung (so viel wie nötig, so wenig wie möglich stimulieren).

Im Rahmen der DDD-Stimulation muss eine untere Grundfrequenz („lower rate limit“, LRL) programmiert werden (ein Unterschreiten lässt der HSM nicht zu) sowie eine maximale Trackingfrequenz („upper rate limit“, URL), also die Frequenz, bis zu der eine 1:1-Überleitung von Vorhof auf die Herzkammern durch den Herzschrittmacher erfolgt. Diese auf den ersten Blick simplen Programmierentscheidungen ziehen aber einige Konsequenzen mit sich. Bei jungen Patient:innen sollte die maximale Trackingfrequenz mit Bedacht gewählt werden, um zu garantieren, dass bei sportlicher Betätigung weiterhin eine adäquate Stimulation erfolgen kann und nicht plötzlich eine Situation einer 2:1-AV-Leitung bei intrinsischem atrioventrikulärem Block (AV-Block) mit schlagartigem Abfall der Herzfrequenz eintritt. Die maximale Herzfrequenz kann aber auch aufgrund von verschiedenen Parametern nicht unendlich erhöht werden, da eine quasi Konkurrenz mit anderen zu programmierenden Zeiten besteht und ggf. kein ausreichend guter Schutz vor einer Interaktion von Vorhof- und Kammer, sog. „cross-talk“ durch Fernfeldsignale oder Rückleitung besteht.

Durch die Programmierung einer Frequenzhysterese wird eine Toleranz z. B. nachts, für ein Unterschreiten der programmierten Grundfrequenz geschaffen und ein späteres Einsetzen des HSM dennoch gewährleistet.

**Figure 2:**
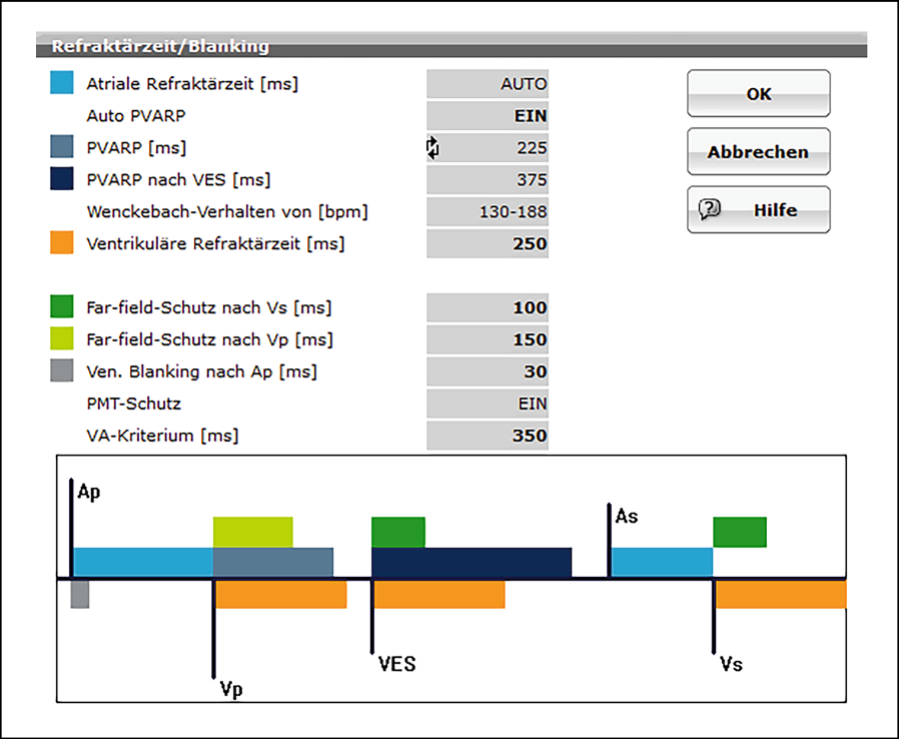

Damit ein Zweikammerschrittmacher möglichst keinerlei Interferenzen (kardiale, Schrittmacher bedingte oder externe) einfängt und verarbeitet, müssen Refraktärzeiten für die Kanäle (A und V) definiert und ggf. angepasst werden. Die Abb. 2 zeigt eine Übersicht über die erweitert programmierbaren Parameter in diesem Zusammenhang, also Refraktärzeiten und Blanking-Perioden. Hier besteht von Seiten der Hersteller eine Vorprogrammierung, diese sollte kritisch geprüft und im Einzelfall entsprechend angepasst werden. Diese Voreinstellung, also „Out-oft-the-box“-Programmierung ist in der Regel DDD mit einer Grundfrequenz von 60/min sowie einer maximalen Frequenz von 130/min.

Neben der Programmierung der minimalen und maximalen Frequenzen ist die Einstellung des AV-Delays ein elementarer Bestandteil der Schrittmachertherapie.

Das AV-Delay ist der zeitliche Abstand zwischen der Vorhof- und Kammererregung, bzw. der Vorhofwahrnehmung und Kammererregung. Das AV-Delay entspricht physiologisch der PQ-Zeit, diese ist außerdem frequenzabhängig. Dies sollte bei der Programmierung auch bedacht werden. Häufig wird außerdem zwischen dem programmierten wahrgenommenen AV-Intervall (SAV) und dem stimulierten AV-Intervall (PAV) unterschieden.

Die Länge der programmierten AV-Zeit spielt nicht nur eine Rolle bei der Unterdrückung von ventrikulärer Stimulation, sondern hat auch Auswirkungen auf die Hämodynamik durch die Dauer der im UKG zu messenden E- und A‑Welle und dem Einfluss auf die diastolische Füllung. Für die Programmierung der hämodynamisch optimalen AV-Zeit, am besten unter direkter echokardiographischer dopplersonographischer Kontrolle des Mitralkappeneinstromprofils unter Messung der E‑ und der A‑Welle kann die Ritter-Formel angewendet werden [12]. Auch über das Oberflächen-EKG ist eine AV-Zeit-Optimierung nach der Koglek-Formel möglich, bei der Zeit vom Ende der P‑Welle zur Spitze der R‑Welle optimiert wird [9].

Um den ventrikulären Stimulationsanteil zu reduzieren, sollte das AV-Delay entsprechend angepasst werden. Liegt z. B. im Ruhe-EKG ein AV-Block I° mit 210 ms Überleitungszeit vor und ist der Herzschrittmacher mit einer AV-Zeit von 160 ms programmiert, so kommt es zu einer 100%igen ventrikulären Stimulation, obwohl ein entsprechender Eigenrhythmus vorhanden ist. Dies gilt es zu vermeiden. Daher sollte im Rahmen der Nachsorge bei entsprechendem ventrikulären Eigenrhythmus ein Blick auf die nativen Leitungszeiten geworfen werden, um eine ideale Einstellung zu finden.

Alle Hersteller haben eigene Algorithmen (AAI-SafeR, VIP, IRS+, MVP, Search-AV sowie RhythmIQ), um eine Reduktion des ventrikulären Stimulationsanteils zu ermöglichen. Eine Verlängerung der AV-Zeit auf > 230 ms (AV-Intervall intrinsischer P‑Welle, SAV) bzw. > 270 ms (AV-Intervall nach atrialer Stimulation, PAV) sollte aber aus den oben genannten Gründen nicht erfolgen [6].

**Figure 3:**
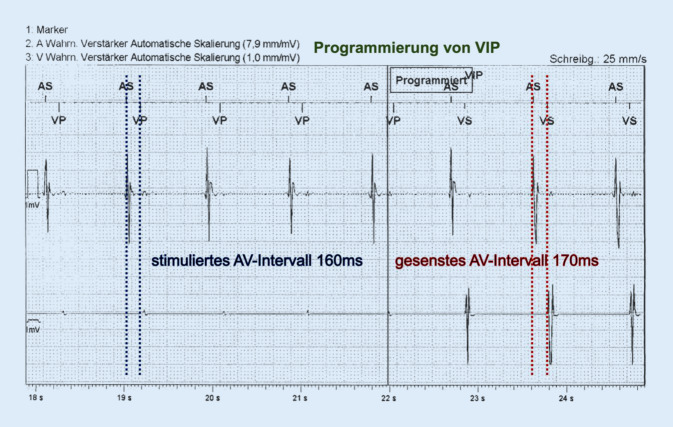

Die Abb. 3 zeigt ein Beispiel, bei dem die Verlängerung des AV-Delays um 10 ms bereits zu einer Reduktion des ventrikulären „pacing“ geführt hat.

## Auftreten von Rhythmusstörungen

Bei Patient:innen mit implantiertem Herzschrittmacher liegen nicht selten neben der intermittierenden oder persistierenden Bradykardie als Grund für die Herzschrittmacherimplantation auch tachykarde Rhythmusstörungen vor. Bei vielen Patient:innen finden sich Vorhofrhythmusstörungen (zumeist Vorhofflimmern, VHF). Im Rahmen der Programmierung ist wichtig, dass das VHF vom Herzschrittmacher als solches erkannt wird, um eine entsprechende automatische Anpassung der Programmierung vornehmen zu können. Um VHF entsprechend richtig erkennen zu können, muss dieses auch im Vorhofkanal ausreichend gut „gesehen werden“. Dies ist dann schwierig, wenn das „sensing“ im Vorhof schon niedrig ist und dann die meist niedrig-amplitudigen Vorhofsignale im Rahmen des VHF nicht erkannt werden. Hier ist es auch wichtig, die Einstellung der Empfindlichkeit zu prüfen, damit eine entsprechende Detektion erfolgen kann.

**Figure 4:**
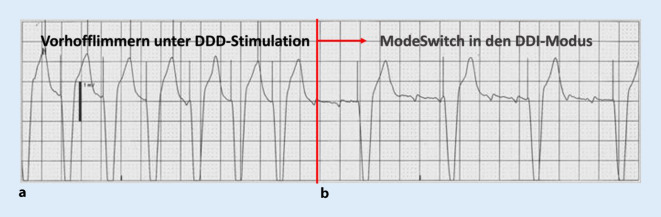

Sobald VHF bzw. eine andere Vorhoftachyarrhythmie vorliegt, sollte eine entsprechende automatische Umstellung in den Mode-Switch erfolgen. Mode-Switch bedeutet eine automatische Umstellung des DDD(R)-Modus in den DDI(R)-Modus, um eine unnötig tachykarde Überleitung des VHF zu vermeiden (Abb. 4). Hier gilt es zu beachten, dass der Mode-Switch dann erfolgt, wenn auch wirklich eine Vorhofrhythmusstörung vorliegt und nicht fälschlicherweise bei z. B. Störsignalen auf der Vorhofelektrode, v. a. Fernfeldsignale aus dem Ventrikel („ventricular far-field oversensing“). Des Weiteren ist die Mode-Switch-Frequenz, d. h. die wahrgenommene Vorhoffrequenz, ab der ein Mode-Switch erfolgen soll, bei vielen Herstellern unterschiedlich vorprogrammiert (meist 170–180/min) und kann den Arrhythmien des Patienten angepasst werden. Bei langsamem Vorhofflattern etwa kann es sinnvoll sein, die Mode-Switch-Frequenz auf 150/min abzusenken. Bei einigen Schrittmachern kann auch eine separate Stimulationsfrequenz während Mode-Switch programmiert werden, was bei AV-Block III° sinnvoll sein kann, z. B. untere Grenzfrequenz 50/min im (normalen) Sinusrhythmus, 70/min bei Vorhofflimmern und Mode-Switch.

Eine weitere Herausforderung für die Programmierung von Zweikammerherzschrittmachern sind regelmäßige Vorhoftachykardien, bei denen der Herzschrittmacher durch die Einstellung der Blanking-Zeiten jede zweite P‑Welle der Vorhoftachykardie „übersieht“ und daraufhin die jeweils andere (= jede 2.) P‑Welle wahrnimmt und auf die Herzkammer überleitet. Hier spricht man von einem „2:1-Lock-in“. Mit den aktuell in den Herzschrittmachern implementierten Algorithmen kommt dieses Phänomen selten vor, bei Auftreten muss aber eine entsprechende Anpassung der AV- (SAV) und Blanking-Zeiten erfolgen, damit die Tachykardie adäquat erkannt wird und ein Mode-Switch erfolgen kann.

Eine Form der Gruppe schrittmachervermittelter Tachykardien (PMT) bei Patient:innen mit Zwei- (und Drei‑)Kammerherzschrittmachern stellen sog. Endless-loop-Tachykardien dar, die vom Herzschrittmacher selbst unterhalten werden. Ein Zweikammerschrittmacher kann auch als implantierte akzessorische Bahn mit ausschließlich antegrader Leitung interpretiert werden. Diese Form der Rhythmusstörung, die für betroffene Patient:innen häufig – ähnlich einer AV-Knoten-Reentry-Tachykardie – sehr unangenehm ist, kann durch verschiedene Mechanismen entstehen (z. B. ventrikuläre Extrasystole mit retrograder VA-Leitung) und unterhält sich von alleine. Die aktuellen Algorithmen der implantierbaren Herzschrittmacher können solche PMT erkennen und selbstständig terminieren. Damit diese nicht auftreten, sollte auf die Einstellung der Refraktärzeiten geachtet werden und z. B. eine Verlängerung der PVARP nach ventrikulärer Extrasystole (sog. „VES-Reaktion“) aktiviert werden.

## HIS-Bundle-Programmierung

Die HIS-Stimulation als physiologischste Form der Herzschrittmachertherapie stellt nicht nur bei der Implantation, sondern auch bei der Nachsorge neue Anforderungen an Ärzte und Material. Diese wiederentdeckte Form der Implantation der Ventrikelelektrode am His-Bundle ist aktuell im Fokus von klinischen Studien und nicht für jeden Patienten indiziert [6, 8].

Aufgrund der Implantation der RV-Elektrode am His-Bundle und dessen elektrischer Beschaffenheit muss bei einem Teil der Patienten mit einer erhöhten Reizschwelle und daher kürzeren Batterielaufzeit gerechnet werden. Durch die Programmierung einer längeren Impulsdauer (in der Regel 1,0 ms) kann bei der zu programmierenden Amplitude „eingespart“ werden. Auch ist auf eine mögliche Änderung des QRS-Komplexes bei unterschiedlichen Amplituden und Impulsdauern zu achten (Reizschwelle immer mit mitlaufendem 12-Kanal-EKG), da direktes, indirektes und nicht-selektives His-Capture ineinander übergehen können. Die Reizschwelle sollte im VVI-Modus mit relativ hoher Frequenz (z. B. 90–100/min) durchgeführt werden, da bei Eigenüberleitung eine ineffektive genauso wie eine effektive selektive His-Bundle-Stimulation aussehen kann (stimulierter und nicht stimulierter QRS sehen identisch aus!).

Automatische Reizschwellenanpassungen sollten bei dieser Form der Stimulation nicht verwendet werden, da sie meist auf Erkennung des Myokard-Captures beruhen und bei selektiver His-Bundle-Stimulation tatsächlich nur das His-Bundle, aber kein Myokard stimuliert wird.

**Figure 5:**
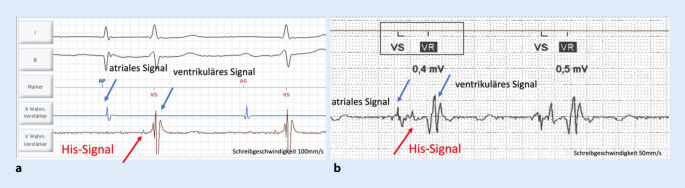

Auch die Einstellung der Sensing-Parameter müssen patientenindividuell erfolgen. Auf dem ventrikulären Kanal sind statt der üblich „isolierten“ R‑Welle, 3 Komponenten mit unterschiedlicher Amplitude abgrenzbar. Das atriale Fernfeldsignal und das His-Bundle-Signal sollten nicht gesehen werden, eine R‑Welle soll aber erkannt werden. Diese gemessenen Werte für das R‑Wellen-Sensing sind in der Regel niedriger als bei konventionell implantierten Herzschrittmacherelektroden, oft im Bereich von 2,0 mV (Abb. 5).

Hier ist eine gezielte Programmierung der Wahrnehmungsschwelle ohne automaische Anpassung (mögliche automatische „Optimierung“ der Einstellung auf das Erkennen des atrialen Fernfeldsignals!) und u. U. auch eine Anpassung von Zeiten wie postatrialem ventrikulärem „blanking“ (PAVB), ventrikulärem „blanking“ (VB) und der ventrikulären Refraktärperiode (VRP) bei ggf. Rückleitung notwendig.

Ist das Herzschrittmachersystem mit einer Back-up-Elektrode im rechten Ventrikel implantiert worden, dies wird z. B. bei höhergradigem AV-Block empfohlen, ist diese in der Regel am RV-Kanal zu finden sowie die His-Elektrode am LV-Kanal [6]. In dieser Konstellation ist auf ein langes, oft maximales V‑V-Delay (≥ 80 ms) zu achten, allerdings nicht > 150 ms. Außerdem sollte der VVT-Modus, der bei Systemen zur kardialen Resynchronisation (CRT) oft nominal aktiviert ist, unbedingt vermieden werden.

Die Programmierung und Nachsorge von implantierten His-Bundle-Elektroden ist aktuell herausfordernd, da die bestehenden Algorithmen zur Nachsorge und Programmierung auf diese Form der Stimulation sowie die veränderten Messwerte und intrakardialen Signale (noch) nicht ausgelegt sind. Weitere Übersichten zur Programmierung im Detail sowie zum Lösen von Programmierproblemen finden sich hier [2, 6–8].

## Fazit für die Praxis

- Eine grundlegende Kenntnis der zu programmierenden Parameter eines Herzschrittmachers ist für alle Kardiolog:innen wichtig.
- Die Programmierung von Herzschrittmachern kann im Einzelfall herausfordernd sein und sollte immer an die individuellen Bedürfnisse des Patienten angepasst werden.
- „Out-of-the-box“-Programmierungen sollten vermieden werden.
- „Cross-talk“ zwischen Vorhof- und Kammerelektrode sollte vermieden werden.
- Eine genaue Kenntnis der herstellerspezifischen Algorithmen ist unabdingbar.
- Die His-Bundle-Stimulation stellt sowohl die nachsorgenden und implantierenden Ärzt:innen als auch die Hersteller vor neue Herausforderungen bei der Programmierung.
